# Anti-Cancer Effects of REIC/Dkk-3-encoding Adenoviral Vector for the Treatment of Non-small Cell Lung Cancer

**DOI:** 10.1371/journal.pone.0087900

**Published:** 2014-02-03

**Authors:** Kazuhiko Shien, Norimitsu Tanaka, Masami Watanabe, Junichi Soh, Masakiyo Sakaguchi, Keitaro Matsuo, Hiromasa Yamamoto, Masashi Furukawa, Hiroaki Asano, Kazunori Tsukuda, Yasutomo Nasu, Nam-Ho Huh, Shinichiro Miyoshi, Hiromi Kumon, Shinichi Toyooka

**Affiliations:** 1 Department of Clinical Genomic Medicine, Okayama University Graduate School of Medicine, Dentistry and Pharmaceutical Sciences, Okayama, Japan; 2 Department of Thoracic Surgery, Okayama University Graduate School of Medicine, Dentistry and Pharmaceutical Sciences, Okayama, Japan; 3 Department of Urology, Okayama University Graduate School of Medicine, Dentistry and Pharmaceutical Sciences, Okayama, Japan; 4 Center for Gene and Cell Therapy, Okayama University Hospital, Okayama, Japan; 5 Innovation Center Okayama for Nanobio-Targeted Therapy (ICONT), Okayama University, Okayama, Japan; 6 Department of Cell Biology, Okayama University Graduate School of Medicine, Dentistry and Pharmaceutical Sciences, Okayama, Japan; 7 Department of Preventive Medicine, Kyusyu University Faculty of Medical Science, Fukuoka, Japan; Virginia Commonwealth University, United States of America

## Abstract

**Objectives:**

REIC/Dkk-3 is down-regulated in a broad range of human cancer cells and is considered to function as a tumor suppressor. We previously reported that REIC/Dkk-3-expressing adenovirus vector (Ad-REIC) induced endoplasmic reticulum (ER) stress and cancer-specific apoptosis in human prostate cancer. In this study, we examined the therapeutic impact of Ad-REIC on non-small cell lung cancer (NSCLC).

**Materials and Methods:**

We examined the anti-tumor effect of Ad-REIC on 25 NSCLC cell lines *in vitro* and A549 cells *in vivo*. Two of these cell lines were artificially established as EGFR-tyrosine kinase inhibitor (TKI) resistant sublines.

**Results:**

Ad-REIC-treatment inhibited the cell viability by 40% or more in 13 (52%) of the 25 cell lines at multiplicity of infection (MOI) of 20 (20 MOI). These cell lines were regarded as being highly sensitive cells. The cell viability of a non-malignant immortalized cell line, OUMS-24, was not inhibited at 200 MOI of Ad-REIC. The effects of Ad-REIC on EGFR-TKI resistant sublines were equivalent to those in the parental cell lines. Here, we demonstrated that Ad-REIC treatment activated c-Jun N-terminal kinase (JNK) in NSCLC cell lines, indicating the induction of ER stress with GRP78/BiP (GRP78) up-regulation and resulting in apoptosis. A single intratumoral injection of Ad-REIC significantly inhibited the tumorigenic growth of A549 cells *in vivo*. As predictive factors of sensitivity for Ad-REIC treatment in NSCLC, we examined the expression status of GRP78 and coxsackievirus and adenovirus receptor (CAR). We found that the combination of the GRP78 and CAR expressional statuses may be used as a predictive factor for Ad-REIC sensitivity in NSCLC cells.

**Conclusion:**

Ad-REIC induced JNK activation and subsequent apoptosis in NSCLC cells. Our study indicated that Ad-REIC has therapeutic potential against NSCLC and that the expression statuses of GRP78 and CAR may predict a potential therapeutic benefit of Ad-REIC.

## Introduction

Lung cancer is the most common cause of death from cancer worldwide, and the metastatic form is a major factor leading to mortality [1]. There are two major histological subtypes of lung cancer: non-small cell lung cancer (NSCLC) and small cell lung cancer. Recent intensive studies have identified causative molecular alterations that have directly led to the development of new therapeutic strategies and have improved patient prognosis [2]. For example, mutations of the epidermal growth factor receptor gene (*EGFR*) are found in approximately 30% of NSCLCs, especially in lung adenocarcinomas, and EGFR-tyrosine kinase inhibitors (TKIs) are particularly effective in these tumors [3], [4]. More recently, crizotinib has been shown to be effective for NSCLCs with an *EML4-ALK* fusion gene [5], [6]. However, the number of patients with these alterations is limited, and little improvement in prognosis has been obtained in NSCLCs without these drug-sensitive alterations. Furthermore, acquired resistance eventually occurs in the majority of *EGFR*-mutant tumors, which had previously responded to EGFR-TKI, after an average of 10 months of treatment [7]. Thus, a new therapeutic modality is needed to improve the clinical outcome of patients with lung cancer.

REIC/Dkk-3, a member of the Dickkopf (Dkk) gene family, is originally found in immortalized cells and has been reported to be a tumor suppressor; its expression is significantly down-regulated in a broad range of cancer cell types, including lung cancer [8]. The heatmap image of messenger RNA (mRNA) expression of *REIC/Dkk-3* gene from the UCSC Cancer Genome Browse, which is freely available public database (https://genome-cancer.ucsc.edu/) (we downloaded the data on July 16 2013), showed that *REIC/Dkk-3* gene expression was reduced in majority of examined samples of both lung adenocarcinomas and squamous cell carcinomas compared with normal lung tissues (Figure S1). In addition, it could be confirmed from a public database that expression of *REIC/Dkk-3* was also low in many NSCLC cell lines (Gene Expression Omnibus repository [http://www.ncbi.nlm.nih.gov/geo, GEO accession GSE4824]). REIC/Dkk-3 is known to interfere with Wnt signaling via Wnt receptors [9], [10] and was previously reported to play a distinct role in the induction of apoptosis and the inhibition of metastasis [11], [12]. The induction of apoptosis in cancer cells is mainly caused by endoplasmic reticulum (ER) stress induced by the overproduction of REIC/Dkk-3 in the cells. ER stress triggers the activation of c-Jun N-terminal kinase (JNK), which is a critical event in apoptosis induced by the overproduction of REIC/Dkk-3 using an adenovirus vector (Ad-REIC) [11], [13]. In our previous studies, we found that Ad-REIC had a therapeutic effect on various types of human cancer, including the prostate, testis, pleura, and breast carcinomas [11], [13]–[15]. Ad-REIC infection and REIC/Dkk-3 protein are also known to up-regulate the anti-tumor immunosystem [16]. Based on preclinical data, a clinical trial using Ad-REIC for human prostate cancer has been ongoing in Japan and the USA (NCT01197209).

In this study, we investigated the therapeutic effect of Ad-REIC on NSCLC cells *in vitro* and *in vivo*. We also examined factors related to the sensitivity of cell lines to Ad-REIC as a step toward the development of customized Ad-REIC therapy for patients with NSCLC.

## Materials and Methods

### Ethics Statement

This study was carried out in strict accordance with the recommendations in the Guide for the Care and Use of Laboratory Animals of the National Institutes of Health. The protocol was approved by the Animal Care and Use Committee of Okayama University (Permit Number: OKU-2012-549). All surgery was performed under ketamine and xylazine anesthesia, and all efforts were made to minimize suffering.

### Cell lines

Sixteen cell lines of human lung adenocarcinoma, 3 squamous cell carcinoma, 3 large cell carcinoma, 1 adenosquamous cell carcinoma, 2 EGFR-TKI-resistant sublines from HCC827 and PC-9 cells (HCC827-GR-high2 and RPC-9), the human mesothelioma cell line MSTO-211H (211H), and the normal human fibroblast cell line OUMS-24 were used in this study (Table 1). The details of cell lines used in this study are described in Method S1. The HCC827-GR-high2 and RPC-9 cell lines were established as described previously [17], [18]. OUMS-24 was established at our institution [19].

**Table 1:** Characteristics and the inhibition rate of cell viability on NSCLC cell lines.

Cell lines	Histological subtypes	Genetic alterations	Inhibition rate (%)			GRP78/Actin ratio (Low/High)	CAR/Actin ratio (Low/High)	Category
			20 MOI	100 MOI	200 MOI			
H2009	AD	*KRAS* mut	60	-	-	0.13 (Low)	0.73 (High)	A
H2228	AD	*EML4-ALK* fusion	60	-	-	0.27 (High)	0.70 (High)	B
HCC827	AD	*EGFR* mut	56	-	-	0.25 (High)	0.66 (High)	B
HCC827-GR-high2	AD	*EGFR* mut	55	-	-	0.12 (Low)	2.12 (High)	A
H2087	AD	*BRAF* mut	55	-	-	0.14 (Low)	1.22 (High)	A
HCC4006	AD	*EGFR* mut	54	-	-	0.15 (Low)	0.60 (High)	A
HCC4011	AD	*EGFR* mut	54	-	-	0.22 (Low)	0.13 (Low)	B
H522	AD	W/t	50	-	-	0.27 (High)	1.55 (High)	B
H157	SQ	*KRAS* mut	50	-	-	0.13 (Low)	0.98 (High)	A
A549	AD	*KRAS* mut	49	-	-	0.07 (Low)	0.55 (High)	A
H838	AD	W/t	47	-	-	0.10 (Low)	1.33 (High)	A
H1299	LC	*NRAS* mut	47	-	-	0.08 (Low)	0.68 (High)	A
H661	LC	W/t	40	-	-	0.42 (High)	1.89 (High)	B
H1819	AD	*HER2* amp	23	46	63	0.46 (High)	1.55 (High)	B
H1993	AD	W/t	22	32	40	0.69 (High)	0.09 (Low)	C
H441	AD	*KRAS* mutation	18	49	61	0.21 (Low)	0.17 (Low)	B
H2170	SQ	W/t	18	28	42	0.65 (High)	0.09 (Low)	C
HCC15	SQ	*HER4* mut	17	21	43	0.18 (Low)	0.12 (Low)	B
H460	LC	*KRAS, PIK3CA* mut	17	57	78	0.98 (High)	0.10 (Low)	C
PC-9	AD	*EGFR* mut	16	24	55	0.20 (Low)	0.16 (Low)	B
H1975	AD	*EGFR* mut	10	45	63	0.41 (High)	0.19 (Low)	C
HCC366	ADSQ	W/t	8	42	54	0.39 (High)	0.08 (Low)	C
RPC-9	AD	*EGFR* mut	4	15	40	0.24 (Low)	0.16 (Low)	B
H358	AD	*KRAS* mut	6	59	73	0.57 (High)	1.21 (High)	B
H3255	AD	*EGFR* mut	3	16	40	0.54 (High)	0.59 (High)	B
211H	MM	-	5	13	46	-	-	-
OUMS-24	NHF	-	5	0	0	-	-	-

NSCLC, non-small cell lung cancer; AD, adenocarcinoma; SQ, squamous cell carcinoma; LC, large cell carcinoma; ADSQ, adeno-squamous cell carcinoma; MM, malignant mesothelioma; NHF, normal human fibroblast; mut, mutation; W/t. wild type; MOI, multiplicity of infection.

### Adenovirus vector carrying REIC/Dkk-3

REIC/Dkk-3 was overexpressed using an adenovirus (Ad-REIC) that we have previously generated [11]. A full-length cDNA of REIC/Dkk-3 was integrated into a cosmid vector pAxCAwt and transferred into an adenovirus vector by the COS-TPC method (Takara Bio, Shiga, Japan). An adenovirus vector carrying LacZ gene (Ad-LacZ) was also used as control [11].

### Cell viability assay

Cells were plated in 96-well plates at a density of 1.5×10^3^ cells/well at 48 h after infection with Ad-LacZ or Ad-REIC at a multiplicity of infection (MOI) of 20, 100, or 200 MOI. Cell viability was evaluated 3 days later using an MTS assay with CellTiter 96 Aqueous One Solution Reagent (Promega, Madison, WI).

### Apoptosis assay

To examine the *in vitro* induction of apoptosis after treatment, we seeded the cells in 6-well plates and incubated them for 24 h. The cells were treated with Ad-LacZ or Ad-REIC at 20 MOI in serum-free medium (500 µL) for 2 h; the medium was then exchanged for fresh complete medium (2 mL). After an additional 48 h of incubation, Hoechst 33342 dye (Sigma-Aldrich, St. Louis, MO) was added to the medium at a concentration of 2 µg/mL, and the cells were incubated in the dark for 10 min. Hoechst 33342 is an intercalating dye that allows the determination of variations in the total chromatin quantity and the degree of chromatin condensation [15]. Using fluorescence microscopy, we identified apoptotic cells by the presence of highly condensed or fragmented nuclei. Apoptotic cells were counted in 5 different fields under microscopic observation.

### Western blot analysis

The detailed protocol for the Western blot analysis is described in Method S1. It was performed under conventional conditions using the following antibodies: rabbit anti-human REIC/Dkk-3 antibody raised in our laboratory [11]; rabbit anti-human GRP78/BiP (GRP78) (ab21685; Abcam, Cambridge, MA); rabbit anti-human SAPK/JNK (#9252) and rabbit anti-human phospho-SAPK/JNK (Thr183/Tyr185; #9251) (Cell Signaling Technology, Beverly, MA); rabbit anti-human coxsackievirus and adenovirus receptor (CAR) (HPA030411; Atlas antibodies, Stockholm, Sweden); and mouse anti-actin (MAB1501; Millipore, Billerica, MA). The following secondary antibodies were used: goat anti-rabbit or anti-mouse IgG-conjugated horseradish peroxidase (Santa Cruz Biotechnology, Santa Cruz, CA). To detect the specific signals, the membranes were examined using ECL plus Western Blotting Detection Reagents (Amersham Biosciences UK Limited, Buckinghamshire, UK). In addition, the band intensities for GRP78, CAR, and actin, representing their expression levels, were measured using ImageQuant TL software (GE Healthcare Bioscience) and quantified by GRP78 or CAR/actin ratio.

### Tumor growth assay in vivo

A549 cells (5×10^6^ in 50 µL of phosphate buffered saline [PBS]) mixed with 50 µL of Matrigel (BD Biosciences, San Jose, CA) were subcutaneously injected into the right flank of adult female BALB/c nu/nu mice (CLEA Japan, Tokyo, Japan). The tumor volume was calculated using the empirical formula V = 1/2×[(the shortest diameter)^2^×(the longest diameter)]. When the tumors had reached approximately 50–100 mm^3^, mice (n = 15) were randomly divided into 3 treatment groups: (a) PBS; (b) Ad-LacZ; and (c) Ad-REIC. Viruses (1×10^9^ pfu) in 100 µL of serum-free medium were administered intratumorally. At the end of experiments, mice were sacrificed after 24-days after the viral injection and tumors were harvested, measured, and photographed.

### Statistical analyses

All data were analyzed using STATA ver.12 (STATA Corp., College Station, TX). Fisher's exact test was applied when appropriate. For a comparison of induction of apoptosis between Ad-REIC-treated and Ad-LacZ-treated A549 cells, a Cochran-Mantel-Haenszel statistics was applied for comparing. Repeated measurement ANOVA was applied for the comparison of xenotransplanted NSCLC tumor sizes among PBS, Ad-LacZ and Ad-REIC. *P*<0.05 was considered significant. All tests were two-sided.

## Results

### Effect of Ad-REIC on NSCLC cell lines

**Figure 1:**
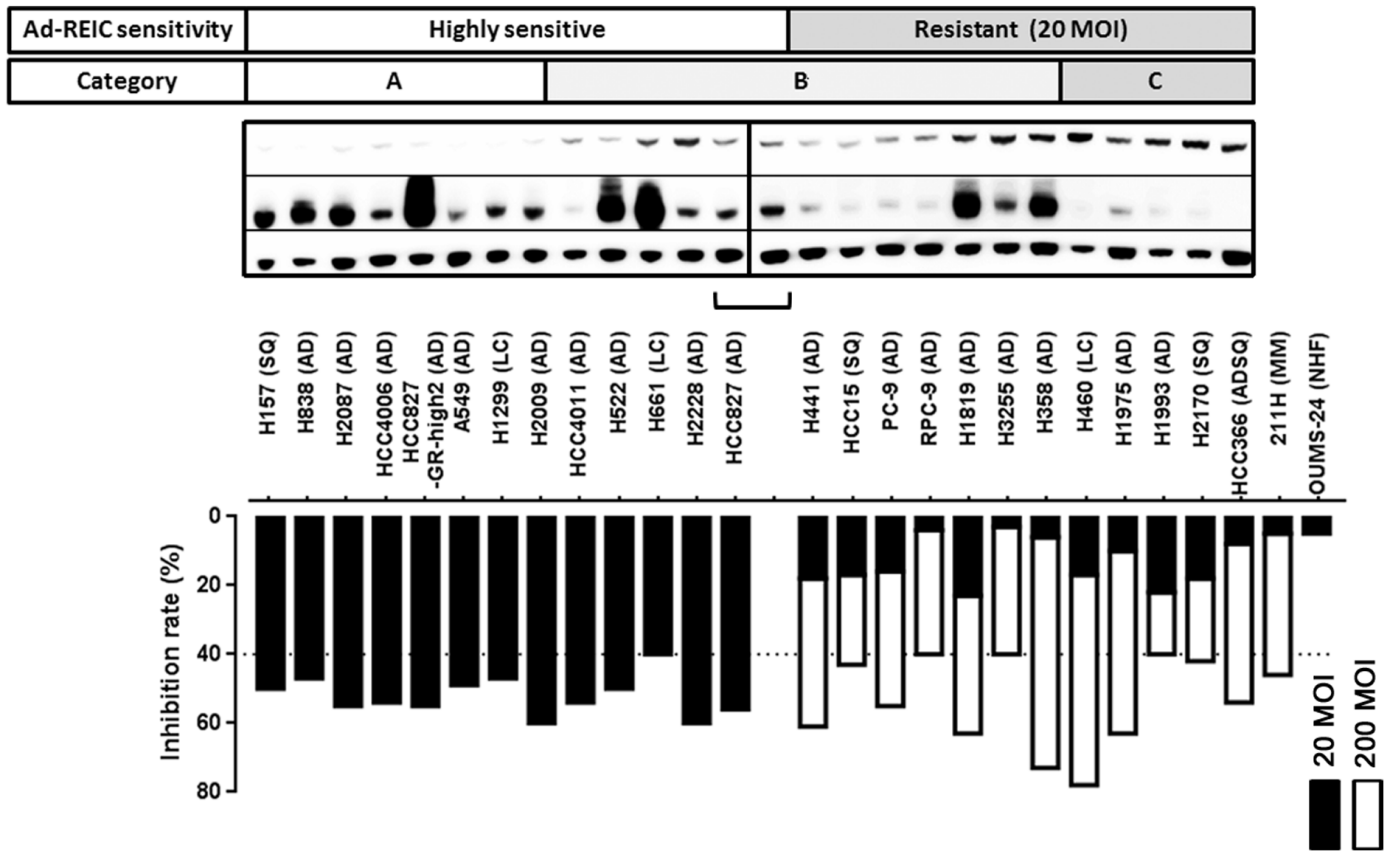
Sensitivity and predictive factors of sensitivity for Ad-REIC treatment in 25 NSCLC cell lines. The inhibition rates of 25 NSCLC cell lines transfected with Ad-REIC compared to Ad-LacZ are shown as black bar in 20 MOI and white bar in 200 MOI. Thirteen cell lines with over 40% inhibition rate in 20 MOI are defined as highly sensitive and 12 cell lines with lower inhibition rate in 20 MOI are defined as resistant. All the resistant cell lines shows over 40% inhibition rate in 200 MOI. The cell lines are classified into 3 categories based on the GRP and CAR protein expression level as follows; category A (low GRP/high CAR), category B (low GRP/low CAR or high GRP/high CAR), category C (high GRP/low CAR). All 8 highly sensitive cell lines were included in category A, and all 5 resistant cell lines were included in category C. Sq; squamous cell carcinoma, AD; adenocarcinoma, LC; large cell carcinoma, ADSQ; adenosquamous cell carcinoma, MM; malignant mesothelioma, NHF; normal human fibroblast.

We examined the inhibition of cell viability using Ad-REIC and an MTS assay. In 13 (52%) of 25 NSCLC cell lines, Ad-REIC treatment at 20 MOI inhibited the cell viability (40%–60% inhibition), compared with Ad-LacZ treatment (Table 1, Figure 1). These cell lines were regarded as highly sensitive to Ad-REIC. In contrast, 12 cell lines (48%) were not inhibited by Ad-REIC treatment at 20 MOI and were regarded as resistant cells. OUMS-24 was not inhibited at 20 or 200 MOI of Ad-REIC. Of note, Ad-REIC treatment at 100 and 200 MOI improved the inhibition of cell viability (100 MOI: 15%–59% inhibition, 200 MOI: 40%–78% inhibition), compared with Ad-LacZ treatment (Table 1). Thus, we defined 20 MOI as a low MOI value and 200 MOI as a high MOI value. For comparison, Ad-REIC treatment was also performed in the human mesothelioma cell line 211H, which we previously reported to be Ad-REIC-sensitive [14]. The 211H was not inhibited at 20 MOI but was inhibited at 200 MOI of Ad-REIC (Table 1). The known molecular characteristics of each cell line are shown in Table 1. The 25 NSCLC cell lines consisted of 8 *EGFR*-mutant, 6 *KRAS*-mutant, 1 *HER4*-mutant, 1 *NRAS*-mutant, 1 *PIK3CA*-mutant, 1 *EML4-ALK* fusion, 1 *HER2*-amplified, and 6 cell lines without gene alterations listed. Nine of the 17 *EGFR*-wild type cell lines were sensitive to Ad-REIC. HCC827 and its resistant subline, HCC827-GR-high2, showed a similar degree of sensitivity to Ad-REIC. No trend in molecular genotype was seen between the sensitive and non-sensitive cell lines. These results suggested that the effect of Ad-REIC does not depend on a known molecular genotype.

**Figure 2:**
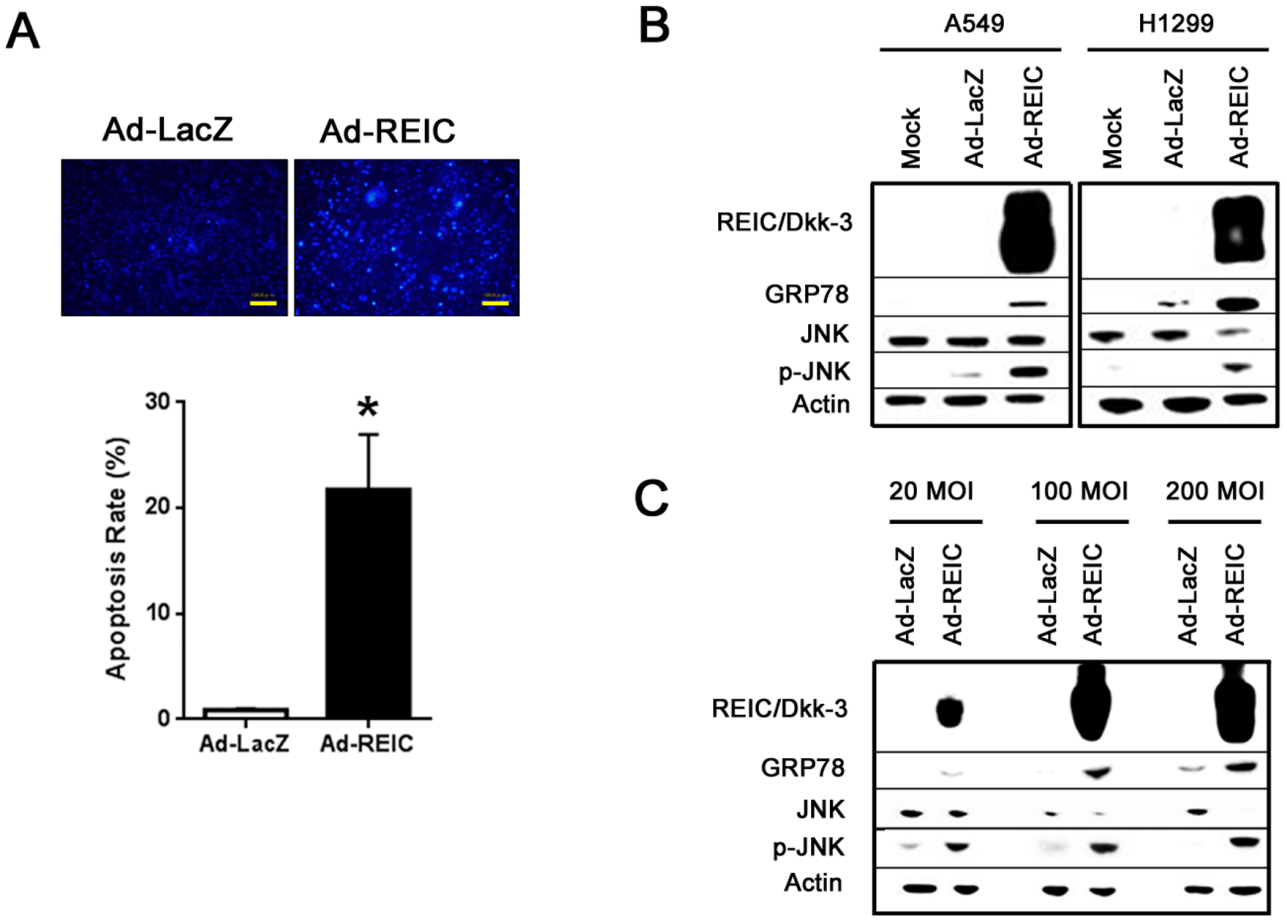
Ad-REIC induced JNK activation and subsequent apoptosis in NSCLC cells. (a) Induction of apoptosis after *in vitro* Ad-REIC treatment as examined in A549 cells using Hoechst 33342 staining. The upper panel indicates the appearance of apoptotic cells after Ad-REIC treatment. The lower panel shows the apoptotic rate of A549 cells after the indicated treatment. A total of 5 different fields were examined under a microscope to determine the apoptotic rate. A significant difference was observed (*p<0.001) between the Ad-LacZ and the Ad-REIC treatment. (bar: 100 µm) (b) Western blot analysis for proteins involved in signal transduction triggered by Ad-REIC. Cells were harvested at 48 h after transfection with Ad-LacZ or Ad-REIC at 20 MOI. (c) H460 cells, which are resistant to adenovirus transduction, were harvested at 48 h after transfection with Ad-LacZ or Ad-REIC at 20, 100, and 200 MOI.

Hoechst 33342 staining was performed in A549 cells to examine the induction of apoptosis. Apoptotic cells were observed in Ad-REIC-treated A549 cells (Figure 2a). The mean rate of apoptosis was 22%, and it was significantly (p<0.001 by Cochran-Mantel-Haenszel test) increased in comparison with the control Ad-LacZ treatment.

The effect of recombinant REIC/Dkk-3 protein on NSCLC cell lines was examined in 7 randomly selected cell lines (NCI-H522, NCI-H611, NCI-H1299, NCI-H1819, NCI-H2009, PC-9, and A549). The MTS assay showed that REIC/Dkk-3 protein did not affect cell viability in the examined cell lines when administered at a concentration ranging from 1 to 200 µg/mL (data not shown).

### Expression of GRP78 and CAR in response to Ad-REIC therapy

As predictive factors of Ad-REIC sensitivity in NSCLC, we examined the expressions of GRP78 and CAR; these expression statuses were correlated with the inhibition of cell viability by Ad-REIC in 13 cell lines. A previous study reported that the overexpression of GRP78 inhibited ER-stress, which may be oppositely correlated with the effect of Ad-REIC. CAR expression is tightly associated with the efficacy of adenovirus infection, which may be positively correlated with the effect of Ad-REIC. Western blotting was performed, and the expression level was quantified as shown in Table 1 and Figure 1. The median (range) of GRP78 and CAR expressions were 0.24 (0.075–0.98) and 0.60 (0.080–2.1), respectively. Based on these data, cells with a GRP78 expression level more than 0.25 were defined as High CRP78 expression, while those with a GRP78 less than 0.24 were defined as Low GRP78 expression. Regarding the CAR, 15 cell lines significantly high level of CAR expression (over 0.50) were defined as High CAR expression, while 10 cell lines those with significantly low level of CAR expression (under 0.20) were defined as Low CAR expression. GRP78 expression was low in 8 of the 13 Ad-REIC-sensitive cells (62%) and in 4 of the 12 Ad-REIC-resistant cells (33%). CAR expression was high in 12 of the 13 Ad-REIC-sensitive cells (92%) and in 3 of the 12 Ad-REIC-resistant cells (25%).

Next, we classified the cell lines into three categories based on the GRP78 and CAR expression statuses; cells with a Low GRP78/High CAR expression were classified as Category A, those with Low GRP78/Low CAR or High GRP78/High CAR expression were classified as Category B, and those with High GRP78/Low CAR expression were classified as Category C. The high sensitive cell rates were 100% in Category A (8 out of 8, 95% confidence interval [CI]: 63–100), 42% in Category B (5 out of 12, 95% CI: 15–72), and 0% in Category C (0 out of 5, 95% CI: 0–52) (Table 2). The categories were significantly associated with the sensitivity to Ad-REIC treatment (p<0.01).

**Table 2:** Ad-REIC sensitivity and categories based on predictive factors.

(n)	Category A (8)	Category B (12)	Category C (5)
**Highly sensitive (13)**	8	5	0
**Resistant in 20 MOI (12)**	0	7	5

### JNK and GRP78 expression in NSCLC cell lines treated with Ad-REIC

A western blotting analysis demonstrated the significant expression of REIC/Dkk-3 protein in 14 NSCLC cell lines treated with Ad-REIC. In 9 cell lines infected with 20 MOI, Ad-REIC treatment resulted in the phosphorylation of JNK and the up-regulation of GRP78 (Figure 2b). In the other 8 cell lines, which were relatively resistant to Ad-REIC, the activation of JNK and GRP78 were observed at higher MOI values (100 and 200 MOI) (Figure 2c).

### Effect of Ad-REIC on NSCLC tumors in a xenotransplantation model

**Figure 3:**
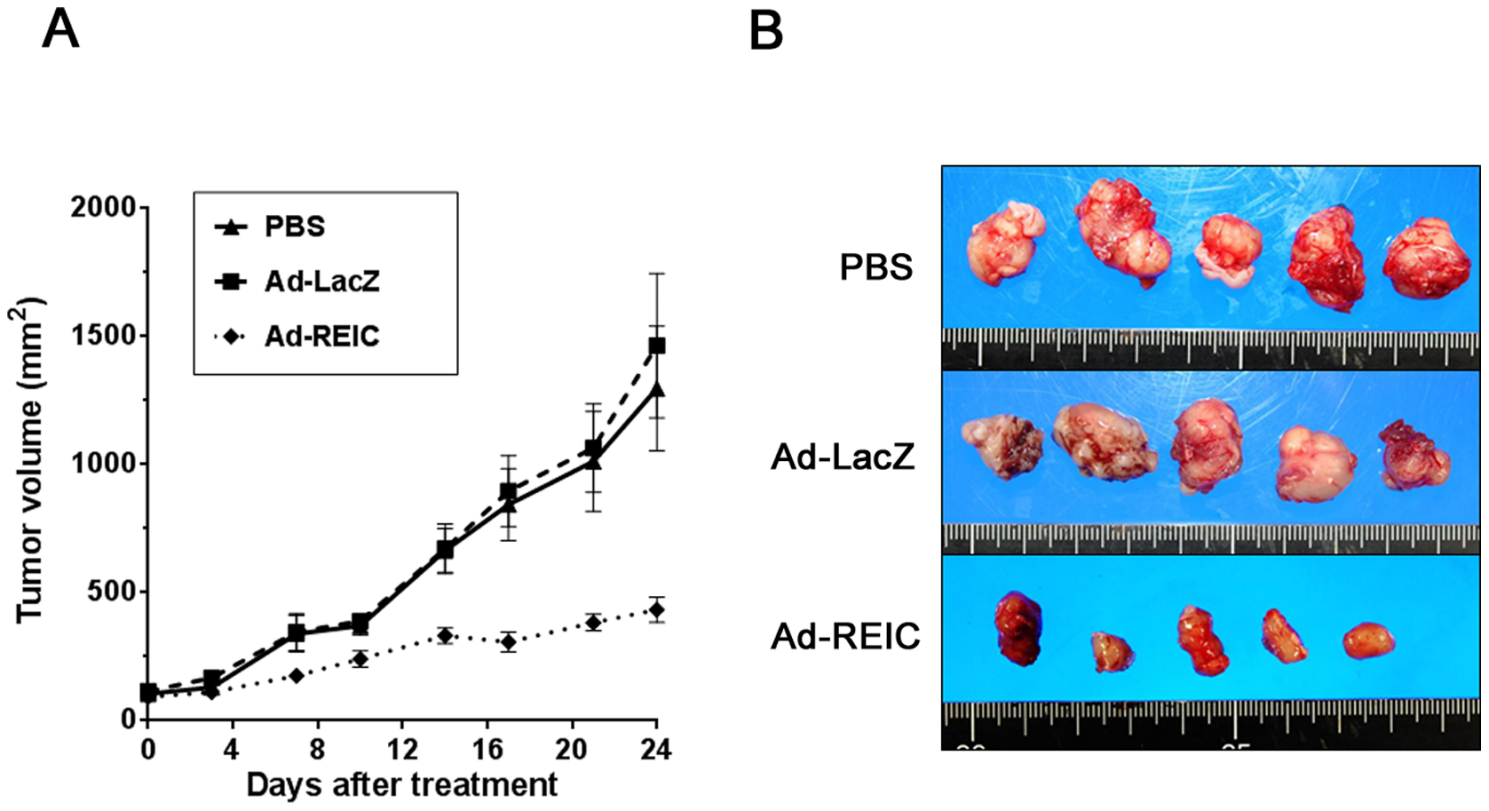
Anti-tumor effect of Ad-REIC treatment on A549 tumor growth *in vivo*. (a) The mean volume of the subcutaneous xenograft tumors was calculated for 5 mice in each group. A significant difference was observed between the results of Ad-REIC and Ad-LacZ treatment (*p<0.001 by repeated measurement of ANOVA). (b) Appearance of the tumors at the time of sacrifice after treatment with PBS, Ad-LacZ, and Ad-REIC.

We investigated the effect of Ad-REIC on the growth of A549 cells *in vivo*. One week after transplantation, when the tumor volume reached 50 to 100 mm^3^, 1×10^9^ plaque-forming units of Ad-REIC or Ad-LacZ in 100 µL of PBS or 100 µL of PBS alone were injected intratumorally. The tumors grew progressively in the PBS and Ad-LacZ treatment groups during the subsequent 24-day observation period. In contrast, the tumor growth in the Ad-REIC treatment group was significantly (p<0.001 by repeated measurement ANOVA) suppressed during the observation period (Figure 3a,b).

## Discussion

In the present study, we found that Ad-REIC was directly effective in more than half of the NSCLC cell lines that were examined, independent of its known driver alterations such as *EGFR* and *KRAS* mutations. An animal xenograft model also showed the therapeutic effect of Ad-REIC. The anti-tumor effect of Ad-REIC depends on ER-stress-mediated JNK activation loaded by the overproduction of REIC/Dkk-3 protein, resulting in the induction of apoptosis [14], [20]. The activation of JNK, which is an essential step in the induction of ER stress and apoptosis by Ad-REIC, was observed at 20 MOI in NSCLC cell lines. On the other hand, the anti-tumor effect of recombinant REIC/Dkk-3 protein was not observed, as in other types of cancers that were previously examined. Originally, REIC/Dkk-3 was identified as a secretory protein and was assumed to exert a physiological function, but its cell surface receptor and its role as a secretory protein have not been identified.

We defined 20 MOI as a low MOI value and 200 MOI as a high MOI value because the normal human fibroblast cell line OUMS-24 was not inhibited at 20 or 200 MOI of Ad-REIC, whereas malignant cell lines were inhibited when the MOI value was elevated to 100 and 200 MOI in cell lines in which Ad-REIC had been ineffective at 20 MOI. In NSCLC, Ad-REIC was effective at a low MOI value in more than half of the cell lines that were tested. Considering the result that 211H was inhibited only at a high MOI value, Ad-REIC might be more effective in NSCLC than in mesothelioma.

Patient selection based on the molecular characteristics of tumor cells is an important theme for maximizing the therapeutic benefit and minimizing adverse effects. For this purpose, we focused on the GRP78 expression and CAR expression levels. GRP78 is a member of the Hsp70 family, which serves as an ER stress-signaling regulator [21]. A previous study showed that the overexpression of GRP78 conferred resistance to a wide variety of chemotherapeutic agents in various kinds of cells [22]. We also showed that the acquired resistance clone of PC-3 cells to Ad-REIC established after repeated exposure to Ad-REIC exhibited a high expression level of GRP78, compared with parental PC-3 cells [13]. Theoretically, Ad-REIC should be effective for tumor cells defined as Category A and not as effective for those defined as Category C. Although sensitive cells in Category B were identified, all 8 cells in Category A responded to Ad-REIC treatment. These results suggested that the expression statuses of GRP78 and CAR in tumors might be useful as biomarkers for customized Ad-REIC therapy in NSCLC while further confirmation is needed by a large scaled investigation using various kinds of cell lines.

As a recent topic of lung cancer treatment, EGFR-TKIs have been shown to be effective for the treatment of *EGFR*-mutant NSCLCs. However, acquired resistance to EGFR-TKIs after TKI treatment is a problem that needs to be overcome. In the current study, our results showed that the effect of Ad-REIC against acquired EGFR-TKI-resistant cells was equal to that against the parental cells, suggesting that Ad-REIC may be useful after the acquisition of resistance to EGFR-TKIs.

Although adenovirus vectors carrying appropriate tumor suppressor genes, such as REIC/Dkk-3, have great potential for cancer gene therapy, they do not exhibit target specificity and therefore may also infect normal cells in the vicinity of cancer cells. The authors reported that the infection of normal human fibroblasts (NHF) with Ad-REIC did not cause the apoptosis of NHF itself, but instead induced the production of interleukin (IL)-7. When Ad-REIC-infected NHF were mixed with untreated cancer cells and the mixture was transplanted into mice, the growth of the cancer cells was significantly suppressed, suggesting an indirect tumor-suppressive effect of Ad-REIC mediated by IL-7 [20]. These findings show that the mis-targeted infection of cancer stroma cells by Ad-REIC activates the immune system through the production of IL-7. In addition, the authors reported that REIC/Dkk-3 protein played a cytokine-like role in monocyte differentiation into dendritic-cell-like features *in vitro* and that the infiltration of CD11c- and CD8-positive (dendritic and killer T cell markers, respectively) cells was observed within the treated tumors *in vivo*. In the experiment using an orthotopic prostate tumor model with pre-established lung metastasis, the number of metastatic lung tumors significantly decreased after the injection of Ad-REIC at the primary tumor site in addition to the inhibition of the growth of orthotopic prostate tumors, suggesting that anti-cancer immune up-regulation by Ad-REIC treatment in primary tumor sites triggered anti-tumor effects even at distant tumor site [16]. These facts strongly suggest that REIC/Dkk-3 shows an indirect anti-tumor effect through the anti-tumor immune system that is an important factor in the treatment of metastatic disease. Because Ad-REIC has both direct and indirect effects on cancer therapy, it may become a powerful therapeutic option as a “one-bullet, two-arms” anti-cancer agent especially for NSCLCs, which often metastasize to other organs.

In regards to clinical usage, because our data suggest that CAR and GRP78 expression statuses in tumor cells predict the responsiveness of Ad-REIC treatment, Ad-REIC treatment should be preferentially performed for patients who are categorized as high sensitive group in early phase of treatment with low dose Ad-REIC. For patients whose tumor cells reveal intermediate or poor effectiveness with low dose Ad-REIC, it should be late phase in their treatment with high dose Ad-REIC. For these patients, cost effectiveness for treatment and clinical outcome should be carefully considered. As for administration strategy, local administration might be preferable rather than systemic administration to minimize the adverse effect in clinical situations. We previously confirmed in mouse model that Ad-REIC could be widely distributed in the bodies after intratumoral local administration, and local administration was effective not only directly but also indirectly through the immune system effect [16], [23]. In addition, intrapleural local administration could be another administration strategy for the patients with malignant pleural effusions. It has been reported that the intrapleural administration of adenoviral-mediated gene therapy is a useful approach for the generation of anti-tumor immune responses in malignant mesothelioma and metastatic pleural effusion in several clinical trials [24], [25].

In conclusion, we demonstrated that Ad-REIC induced JNK activation and subsequent apoptosis in NSCLC cells irrespective of the type of known molecular alterations or the sensitivity to EGFR-TKI. The present study suggests that Ad-REIC has a therapeutic potential for NSCLC, and the expression statuses of GRP78 and CAR may be a predictor of Ad-REIC therapy.

## Supporting Information

Figure S1 **The heatmap image of mRNA expression of** ***REIC/Dkk-3*** **gene.** The mRNA expression level of *REIC/Dkk-3* gene was obtained from the UCSC Cancer Genome Browse, which is freely available public database (https://genome-cancer.ucsc.edu/) (we downloaded the data on July 16 2013), showed that *REIC/Dkk-3* gene expression was reduced in majority of examined samples of both (a) lung adenocarcinomas and (b) squamous cell carcinomas, compared with normal lung tissues.
(PDF)

Method S1 **Supporting information for cell lines and Western blot analysis.**
(DOC)

## References

[1] Jemal A, Siegel R, Ward E, Hao Y, Xu J, et al (2009) Cancer statistics, 2009. CA Cancer J Clin 59: 225–249. 19474385 10.3322/caac.20006

[2] Larsen JE, Cascone T, Gerber DE, Heymach JV, Minna JD (2011) Targeted therapies for lung cancer: clinical experience and novel agents. Cancer J 17: 512–527. 22157296 10.1097/PPO.0b013e31823e701a PMC3381956

[3] Shigematsu H, Lin L, Takahashi T, Nomura M, Suzuki M, et al (2005) Clinical and biological features associated with epidermal growth factor receptor gene mutations in lung cancers. J Natl Cancer Inst 97: 339–346. 15741570 10.1093/jnci/dji055

[4] Tokumo M, Toyooka S, Kiura K, Shigematsu H, Tomii K, et al.. (2005) The relationship between epidermal growth factor receptor mutations and clinicopathologic features in non-small cell lung cancers. Clin Cancer Res. 15709185

[5] Kwak EL, Bang YJ, Camidge DR, Shaw AT, Solomon B, et al (2010) Anaplastic lymphoma kinase inhibition in non-small-cell lung cancer. N Engl J Med 363: 1693–1703. 20979469 10.1056/NEJMoa1006448 PMC3014291

[6] Shaw AT, Yeap BY, Solomon BJ, Riely GJ, Gainor J, et al (2011) Effect of crizotinib on overall survival in patients with advanced non-small-cell lung cancer harbouring ALK gene rearrangement: a retrospective analysis. Lancet Oncol 12: 1004–1012. 21933749 10.1016/S1470-2045(11)70232-7 PMC3328296

[7] Oxnard GR, Janjigian YY, Arcila ME, Sima CS, Kass SL, et al (2011) Maintained sensitivity to EGFR tyrosine kinase inhibitors in EGFR-mutant lung cancer recurring after adjuvant erlotinib or gefitinib. Clin Cancer Res 17: 6322–6328. 21831955 10.1158/1078-0432.CCR-11-1080 PMC3186869

[8] Tsuji T, Miyazaki M, Sakaguchi M, Inoue Y, Namba M (2000) A REIC gene shows down-regulation in human immortalized cells and human tumor-derived cell lines. Biochem Biophys Res Commun 268: 20–24. 10652205 10.1006/bbrc.1999.2067

[9] Krupnik VE, Sharp JD, Jiang C, Robison K, Chickering TW, et al (1999) Functional and structural diversity of the human Dickkopf gene family. Gene 238: 301–313. 10570958 10.1016/s0378-1119(99)00365-0

[10] Mao B, Wu W, Davidson G, Marhold J, Li M, et al (2002) Kremen proteins are Dickkopf receptors that regulate Wnt/beta-catenin signalling. Nature 417: 664–667. 12050670 10.1038/nature756

[11] Abarzua F, Sakaguchi M, Takaishi M, Nasu Y, Kurose K, et al (2005) Adenovirus-mediated overexpression of REIC/Dkk-3 selectively induces apoptosis in human prostate cancer cells through activation of c-Jun-NH2-kinase. Cancer Res 65: 9617–9622. 16266978 10.1158/0008-5472.CAN-05-0829

[12] Edamura K, Nasu Y, Takaishi M, Kobayashi T, Abarzua F, et al (2007) Adenovirus-mediated REIC/Dkk-3 gene transfer inhibits tumor growth and metastasis in an orthotopic prostate cancer model. Cancer Gene Ther 14: 765–772. 17599093 10.1038/sj.cgt.7701071

[13] Tanimoto R, Abarzua F, Sakaguchi M, Takaishi M, Nasu Y, et al (2007) REIC/Dkk-3 as a potential gene therapeutic agent against human testicular cancer. Int J Mol Med 19: 363–368. 17273781

[14] Kashiwakura Y, Ochiai K, Watanabe M, Abarzua F, Sakaguchi M, et al (2008) Down-regulation of inhibition of differentiation-1 via activation of activating transcription factor 3 and Smad regulates REIC/Dickkopf-3-induced apoptosis. Cancer Res 68: 8333–8341. 18922905 10.1158/0008-5472.CAN-08-0080

[15] Kawasaki K, Watanabe M, Sakaguchi M, Ogasawara Y, Ochiai K, et al (2009) REIC/Dkk-3 overexpression downregulates P-glycoprotein in multidrug-resistant MCF7/ADR cells and induces apoptosis in breast cancer. Cancer Gene Ther 16: 65–72. 18654608 10.1038/cgt.2008.58

[16] Watanabe M, Kashiwakura Y, Huang P, Ochiai K, Futami J, et al (2009) Immunological aspects of REIC/Dkk-3 in monocyte differentiation and tumor regression. Int J Oncol 34: 657–663. 19212670 10.3892/ijo_00000191

[17] Shien K, Toyooka S, Yamamoto H, Soh J, Jida M, et al (2013) Acquired Resistance to EGFR Inhibitors Is Associated with a Manifestation of Stem Cell-like Properties in Cancer Cells. Cancer Res 73: 3051–3061. 23542356 10.1158/0008-5472.CAN-12-4136 PMC4506773

[18] Kobayashi N, Toyooka S, Soh J, Yamamoto H, Dote H, et al (2012) The anti-proliferative effect of heat shock protein 90 inhibitor, 17-DMAG, on non-small-cell lung cancers being resistant to EGFR tyrosine kinase inhibitor. Lung Cancer 75: 161–166. 21767894 10.1016/j.lungcan.2011.04.022

[19] Bai L, Mihara K, Kondo Y, Honma M, Namba M (1993) Immortalization of normal human fibroblasts by treatment with 4-nitroquinoline 1-oxide. Int J Cancer 53: 451–456. 8428798 10.1002/ijc.2910530317

[20] Sakaguchi M, Kataoka K, Abarzua F, Tanimoto R, Watanabe M, et al (2009) Overexpression of REIC/Dkk-3 in normal fibroblasts suppresses tumor growth via induction of interleukin-7. J Biol Chem 284: 14236–14244. 19279003 10.1074/jbc.M808002200 PMC2682872

[21] Kaufman RJ (2002) Orchestrating the unfolded protein response in health and disease. J Clin Invest 110: 1389–1398. 12438434 10.1172/JCI16886 PMC151822

[22] Li J, Lee AS (2006) Stress induction of GRP78/BiP and its role in cancer. Curr Mol Med 6: 45–54. 16472112 10.2174/156652406775574523

[23] Sugimoto M, Watanabe M, Kaku H, Li SA, Noguchi H, et al (2012) Preclinical biodistribution and safety study of reduced expression in immortalized cells/Dickkopf-3-encoding adenoviral vector for prostate cancer gene therapy. Oncol Rep 28: 1645–1652. 22941469 10.3892/or.2012.2001

[24] Sterman DH, Recio A, Carroll RG, Gillespie CT, Haas A, et al (2007) A phase I clinical trial of single-dose intrapleural IFN-beta gene transfer for malignant pleural mesothelioma and metastatic pleural effusions: high rate of antitumor immune responses. Clin Cancer Res 13: 4456–4466. 17671130 10.1158/1078-0432.CCR-07-0403

[25] Sterman DH, Recio A, Haas AR, Vachani A, Katz SI, et al (2010) A phase I trial of repeated intrapleural adenoviral-mediated interferon-beta gene transfer for mesothelioma and metastatic pleural effusions. Mol Ther 18: 852–860. 20068553 10.1038/mt.2009.309 PMC2862532

