# Supplementary figures and images for: Anti-Cancer Effects of REIC/Dkk-3-encoding Adenoviral Vector for the Treatment of Non-small Cell Lung Cancer
Source: PLoS One. 2014 Feb 3;9(2):e87900. doi: 10.1371/journal.pone.0087900 (PMC3912155; doi:10.1371/journal.pone.0087900)

**Figure. S1a**

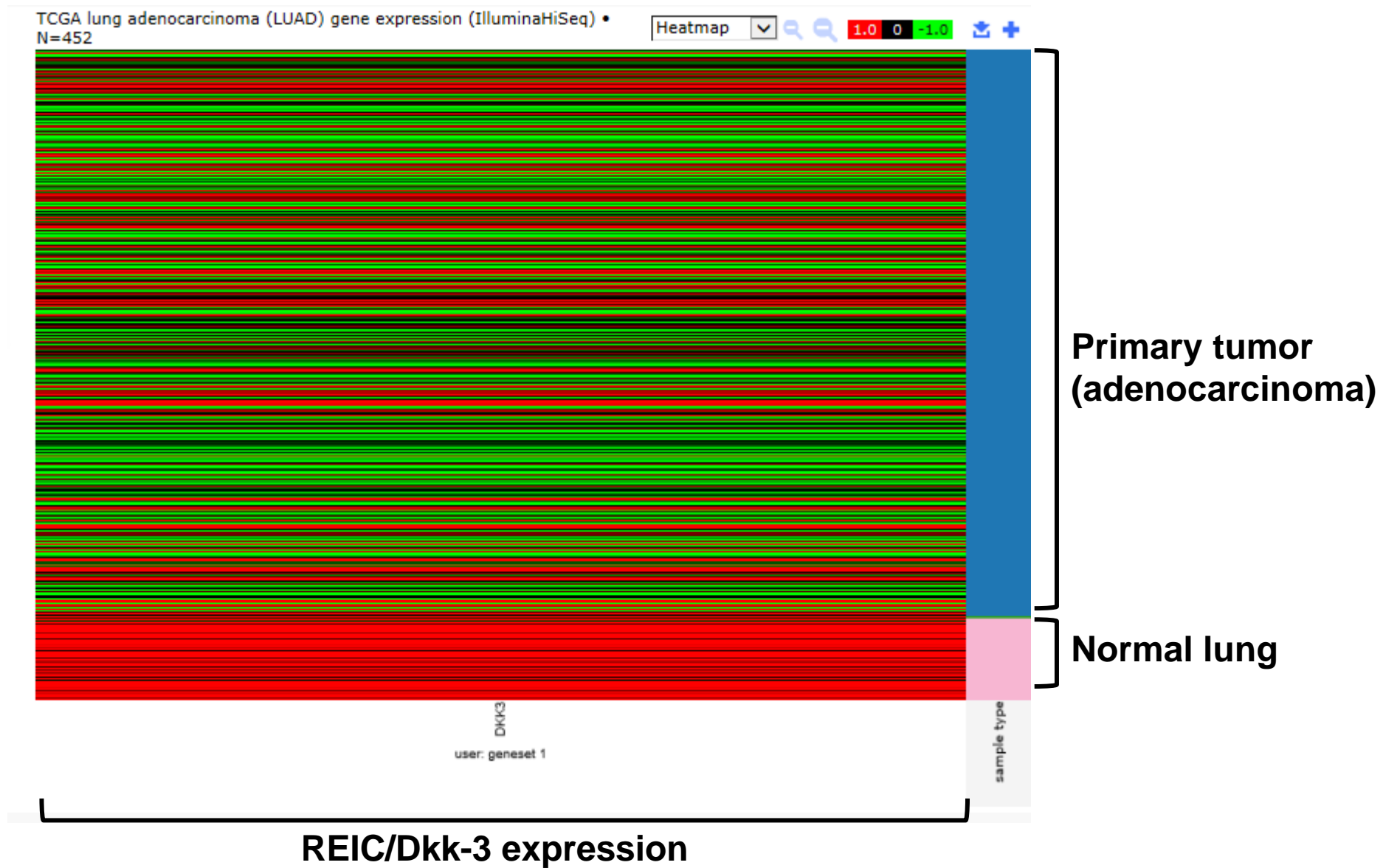

**Figure. S1b**

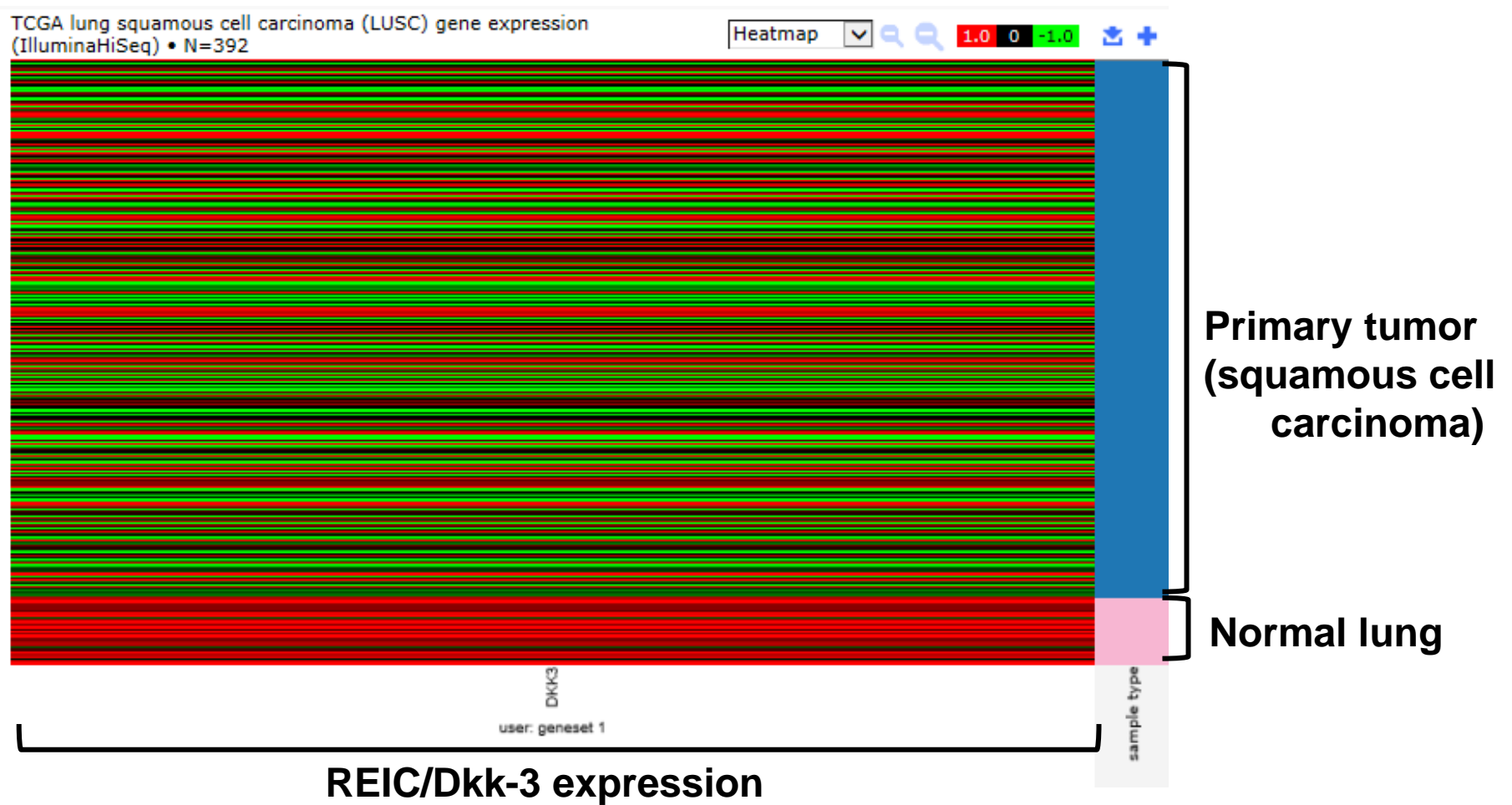

Supplement: Figure S1 — The heatmap image of mRNA expression of REIC/Dkk-3 gene. The mRNA expression level of REIC/Dkk-3 gene was obtained from the UCSC Cancer Genome Browse, which is freely available public database (https://genome-cancer.ucsc.edu/) (we downloaded the data on July 16 2013), showed that REIC/Dkk-3 gene expression was reduced in majority of examined samples of both (a) lung adenocarcinomas and (b) squamous cell carcinomas, compared with normal lung tissues. (PDF) [file pone.0087900.s001.pdf]
