# Supplementary material for: Anti-Cancer Effects of REIC/Dkk-3-encoding Adenoviral Vector for the Treatment of Non-small Cell Lung Cancer
Source: PLoS One. 2014 Feb 3;9(2):e87900. doi: 10.1371/journal.pone.0087900 (PMC3912155; doi:10.1371/journal.pone.0087900)
Supplement: Method S1 — Supporting information for cell lines and Western blot analysis. (DOC) [file pone.0087900.s002.doc]

**Supplementary method S1**

***a. Cell lines***

Sixteen cell lines of human lung adenocarcinoma (NCI-H358 [H358], NCI-H441 [H441], NCI-H522 [H522], NCI-H838 [H838], NCI-H1819 [H1819], NCI-H1975 [H1975], NCI-H1993 [H1993], NCI-H2009 [H2009], NCI-H2087 [H2087], NCI-H2228 [H2228], NCI-H3255 [H3255], HCC827, HCC4006, HCC4011, PC-9, A549), 3 cell lines of human squamous cell carcinoma (NCI-H157 [H157], NCI-H2170 [H2170], HCC15), 3 cell lines of human large cell carcinoma (NCI-H460 [H460], NCI-H661 [H661], NCI-H1299 [H1299]), 1 cell line of human adenosquamous cell carcinoma (HCC366), 2 EGFR-TKI-resistant sublines from HCC827 and PC-9 cells (HCC827-GR-high2 and RPC-9), the human mesothelioma cell line MSTO-211H (211H), and the normal human fibroblast cell line OUMS-24 were used in this study. Cell lines with the prefix NCI-H- (abbreviated as H-) and HCC- were kindly provided by Dr. Adi F. Gazdar (Hamon Center for Therapeutic Oncology Research and Department of Pathology, University of Texas Southwestern Medical Center at Dallas, Dallas, TX). PC-9 was obtained from Immuno-Biological Laboratories (Takasaki, Gunma, Japan). A549 and 211H were obtained from the American Type Culture Collection (Manassas, VA). All the cell lines except for OUMS-24 were maintained in RPMI 1640 medium (Sigma-Aldrich, St. Louis, MO) supplemented with 10% fetal bovine serum (FBS) and incubated at 37°C in a humidified atmosphere with 5% CO_2_. The OUMS-24 cell line was maintained in Dulbecco’s modified minimum Eagle’s medium (Sigma-Aldrich) with 10% FBS.

***b. Western blot analysis***

Cells were grown to 80% confluence and harvested in lysis buffer (20 mmol/L Tris-HCl [pH 7.5], 150 mmol/L NaCl, 1 mmol/L Na_2_EDTA, 1 mmol/L EGTA, 1% Triton, 2.5 mmol/L sodium pyrophosphate, 1 mmol/L beta-glycerophosphate, 1 mmol/L Na_3_VO_4_, 1 μg/mL leupeptin) (Cell Signaling Technology, Beverly, MA) supplemented with Complete, Mini (Roche, Basel, Switzerland) to extract the proteins. A total of 20 µg of protein was separated using SDS-PAGE and was transferred to PVDF membranes. The proteins on the membranes were incubated overnight at 4°C with the primary antibodies. Following methods are as described in the manuscript.
